# Surgical treatment of the bony mallet thumb: a case series and literature review

**DOI:** 10.1007/s00402-021-04333-w

**Published:** 2022-01-15

**Authors:** Tobias Kastenberger, Peter Kaiser, Stefan Benedikt, Kerstin Stock, Magdalena Eigl, Gernot Schmidle, Rohit Arora

**Affiliations:** Department of Orthopaedics and Traumatology, Medical University of Innsbruck, Anichstr. 35, 6020 Innsbruck, Austria

**Keywords:** Fracture, Tendon, Avulsion, Thumb, Mallet

## Abstract

**Introduction:**

The aim of this study was to provide a more precise statement on the outcome after surgical treatment of a bony mallet thumb and possibly give a treatment recommendation regarding the surgical fixation method.

**Patients and methods:**

All patients (*n* = 16) who underwent a surgical treatment for an acute bony mallet thumb fracture between January 2006 and July 2019 were enrolled. The surgical method, complications, the range of motion, tip pinch, lateral key pinch, overall grip strength, visual analog score, Disability of the Arm, Shoulder and Hand Score, Mayo Wrist Score, Patient-Rated Wrist Evaluation Score, Buck-Gramcko Score and radiologic parameters were evaluated. Further, a comprehensive literature search on PubMed was conducted covering a period from 1956 to 2021 to include all possible matching articles on the treatment of the bony mallet thumb (*n* = 21 articles).

**Results:**

Surgical treatment was very inhomogenous including indirect and direct K-wire fixation, screw fixation, plate fixation and anchor fixation methods. The IP joint range of motion and thumb strength ranged from 66 to 94% in comparison to the contralateral side. An open reduction led to worse functional scores compared to a closed reduction. Treatment methods in the literature were also very inhomogenous with a very low patient count, often even pooling data of bony mallet thumb fractures with bony mallet finger fractures. The risk for infection was higher in K-wire fixation methods than in open reduction and internal fixation methods.

**Conclusion:**

The evidence for the best treatment of a bony mallet thumb fracture is low. On one hand the functional outcome can be inferior using an open reduction approach, but on the other hand, K-wire fixation methods with a closed reduction approach showed a higher risk for infection. Future multi-center research must be conducted to find the best treatment procedure for the best outcome of the patient.

**Supplementary Information:**

The online version contains supplementary material available at 10.1007/s00402-021-04333-w.

## Introduction

An avulsion injury of the extensor tendon of the distal interphalangeal (DIP) joint is also called “bony mallet injury/deformity” or “mallet fracture”. A mallet injury to the thumb is referred to as a “mallet thumb” and occurs quite rare [6, 41], especially as an avulsion fracture. This injury to the thumb differs in some features compared to the other phalanges. The extensor pollicis tendon has functionally a greater extensor strength, greater excursion and a stronger tendency for retraction in the event of injury compared with the extensor digitorum tendon. The tendon is also thicker providing a better support for possible sutures. Also, immobilization of the interphalangeal (IP) joint of the thumb alone is not sufficient to relax the tendon in the case of a lesion. Additionally, difference in the tendon attachments and a tighter capsule may limit IP subluxation due to potentially more stability than the DIP joints [5, 29, 31].

The treatment of a bony mallet finger can be conservatively using different kinds of splints [30, 38, 39] or surgically using different fixation methods [9, 14, 21, 22, 40]. Most surgeons recommend surgery for injuries involving more than one third of the articular surface and those with subluxation or displacement [20, 21]. Regarding the bony mallet thumb injury, only few reports with a low level of evidence have been published using different surgical and non-operative fixation methods (two extension block K-wires [25], a hook plate [27, 36, 40], a transverse mini-plate [42], biodegradable devices [1, 28], an Ishiguro extension block technique [29], a direct K-wire pinning [9, 33], compression fixation pins [43], an extension block pinning with direct pinning [13], a pull-out wire fixation [17, 44], a screw fixation [2, 8, 12], tension band wiring [4], non-operative fixation [15], K-wire fixation with sutures [16] or K-wire, cast, splint, suture and screw fixation methods [31]).

Because the occurrence of a bony mallet thumb is a very rare condition, literature reports are limited to a few case reports or series (Table 1). Therefore, the evidence for the best treatment is scarce and the surgical outcome cannot be predicted sufficiently with regard to literature.

**Table 1:** Functional outcome parameters

Outcome parameter	Value
IP joint extension	22° (SD 12°, range 0–45°)/66% of contralateral side
IP joint flexion	54° (SD 22°, range 26–94°)/78% of contralateral side
Total IP joint range of motion	76° (SD 30°, range 32–121°)/74% of contralateral side
Kapandji grade	8.6 (SD 2.3, range 1–10)
Tip pinch	4.5 kg (SD 2.3, range 0.6–8.1 kg)/86% of contralateral side
Lateral key pinch	6.4 kg (SD 2.9, range 2.0–12.8 kg)/81% of contralateral side
Overall grip strength	35.6 kg (SD 15.4, range 3.7–62.3 kg)/94% of contralateral side
VAS at rest	0 (SD 0)
VAS under load/at work	1.4 (SD 1.8, range 0–5)
DASH score	12.3 (SD 17.5, range 0–53.3)
PRWE score	11.9 (SD 17.4, range 0–61)
MWS score	78.1 (SD 11.4, range 50–90)
Buck–Gramcko score	12.8 (SD 2.9, range 6–15)

The aim of this study was to provide a more precise statement on the potential patient outcome after surgical treatment of a bony mallet thumb and possibly give a treatment recommendation regarding the surgical fixation method.

## Patients and methods

All patients above the age of 18 who underwent a surgical treatment for an acute bony mallet thumb fracture between January 2006 and July 2019 were enrolled in this study. Approval to conduct this follow-up study was obtained from the local ethical review board (1211/2020). Informed consent was obtained from all individual participants included in the study.

Twenty one patients were identified who received a primary surgical treatment for an acute bony mallet thumb fracture. Sixteen patients (13 males, 3 females, 21–86 years, mean age 48.8 years; 15 right-handed, 1 ambidextrous) were willing to take part in this follow-up study, which corresponds to a follow-up rate of 76%. The remaining five patients were either unwilling to participate (*n* = 3) or uncontactable (*n* = 2). All patients were contacted via letter and telephone and invited to return into the clinic to conduct a clinical and radiologic follow-up examination.

The objective clinical measurement parameters were the active range of motion (ROM) and the tip pinch, lateral key pinch and overall grip strength for both sides. The range of motion of the patient’s interphalangeal joint of the thumb was measured using the “Goniometer N400” (Biometrics Ltd, Newport, UK). The Kapandji scoring (0–10) was used to assess the amount of opposition of the thumb. Grip strength was assessed with the “Dynamometer G200” (Biometrics Ltd, Newport, UK). The patients were asked to squeeze the dynamometer three times in a row for strength assessment. The mean out of the three measurements was used for calculations.

To evaluate the subjective functional outcome, the patients were asked to reply to a German translation of the “Disability of the Arm, Shoulder and Hand Score” (DASH), the “Mayo Wrist Score” (MWS), the “Patient-Rated Wrist Evaluation Score” (PRWE), the Buck-Gramcko Score and to evaluate their pain level on the visual analog score (VAS), while resting and under load with 0 meaning no pain and 10 meaning the most severe pain.

Radiologic follow-up consisted of a dorsopalmar and lateral radiographs of the interphalangeal joint of the thumb of the affected and uninjured side. Initial radiographs and follow-up radiographs were assessed for a bone union, intraarticular steps or gaps, fragment size and count, interphalangeal joint extension angle, joint subluxation, osteoarthritis (cysts, osteophytes, narrowed joint space) and non-union.

Further data were collected from the medical record (accident mechanism, surgical date and method, subsequent operations, duration of splinting, implant removal and complications) or the anamnesis (handedness, profession learned and practiced preoperatively, change of profession due to the injury and the execution of occupational therapy postoperatively).

Due to the small sample size, non-parametric test (Mann–Whitney *U*-test, Chi-square test, Fisher’s exact test) were used for statistical calculations. Results are presented using descriptive statistics which were calculated with SPSS version 23 (IBM, Armonk, USA). Statistical significance was set at *p* ≤ 0.05.

Further, a comprehensive literature search on PubMed was conducted covering a period from 1956 to 2021 to include all possible matching articles on the treatment of the bony mallet thumb. The search was performed using the search string “mallet” AND “thumb” or “mallet” AND “fracture”. One authors screened the published studies independently by the title and the abstract. All articles which were assessed for eligibility were screened for the word “thumb” within the full-text. All articles which did not include any bony mallet thumb injury were excluded. Only publications reporting on or including a bony mallet thumb injury were included in the qualitative synthesis independent of their quality or study design.

## Results

### Case series

Five patients were blue collar workers, eight white collar workers and three retirees. Two injuries occurred in a motorcycle accident, two after a fall from a bicycle, three while slipping and falling, three while participating in sport activities and three occurred because a heavy object fell on the thumb. The thumb got caught in one patient and two patients were not able to recall the specific accident. The non-dominant side was affected in 13 cases (80%).

The mean age at the time of surgery was 41 years (SD 22, range 15–78 years). The mean time between the accident and the surgery was 3.8 days (SD 5.3, range 0–18 days) with 13 patients being treated within 4 or less days. The mean time until follow-up was 90 months (17–175 months).

**Fig. 1:**
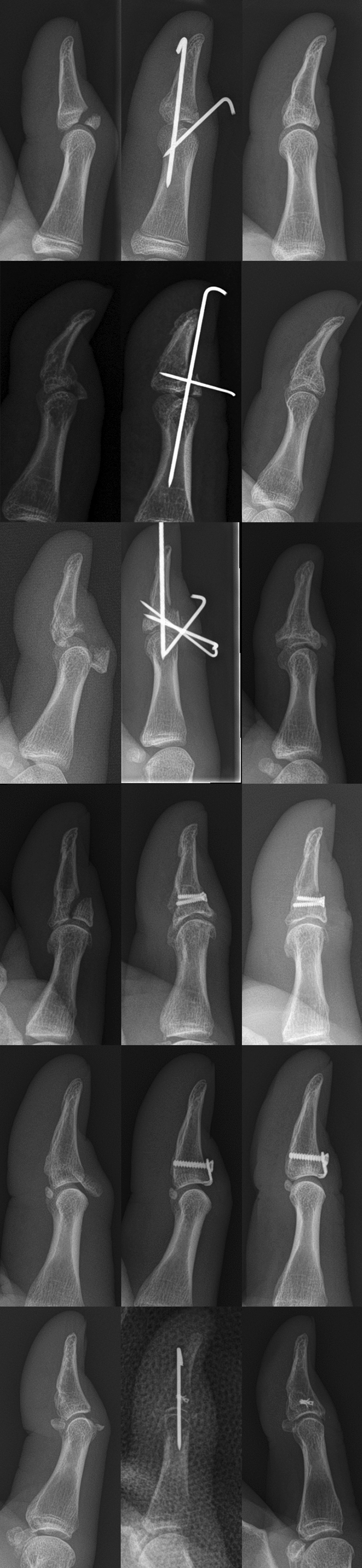
Radiographs of different treatment methods of a bony mallet thumb fracture (after injury, after surgery and at final follow-up)

Eleven patients showed two, four patients three and one patient multiple fracture fragments. According to the Doyle classification, one patient was graded I, six patients were grade IVb and nine patients IVc. Two patients had an open injury while the remaining sustained a closed fracture. The mean fracture fragment size was 5.3 (SD 2.8, range 1.0–10.8) × 5.6 (SD 3.8, range 1.4–17.7) mm. In one patient the fracture consisted of multiple fragments that extended through the entire width of the phalanx and could not be precisely delineated. Therefore, fragment size and number could not be specified in this patient, who was treated with a direct K-wire fixation without transfixation. The other patients were either treated by means of an indirect K-wire fixation (*n* = 5/Ishiguro technique), a direct K-wire fixation (*n* = 4), a screw fixation (*n* = 4), a plate fixation (*n* = 1) or an anchor fixation with a K-wire transfixation (*n* = 1). An IP joint transfixation was conducted in nine patients (56%; five indirect K-wire fixations, two direct K-wire fixations, one screw fixation, one anchor fixation). Three screw fixations, three direct K-wire fixations and one plate fixation were not treated with a transfixation. Examplary cases are shown in Fig. 1.

One patient had a K-wire fixation of the index finger on the same hand. An additional injury to the contralateral side was present in two patients (one Bennett fracture and one fracture of the middle phalanx of the fifth digit).

A splint was worn for a mean of 34 days (SD 8, range 25–51 days). One patient removed the splint on his own and therefore no data on the duration of splinting were noted.

Occupational therapy was performed by all but four patients. The duration could not be sufficiently recorded retrospectively.

Functional outcome parameters are presented in Table 1.

The collateral ligaments were stable in all cases except one case treated with a screw fixation with an increased ulnar distension that was not symptomatic in daily life. Five patients reported a reduced sensitivity of the tip of the thumb or around the scar, which did not bother any patient.

Eleven patients were satisfied with the outcome, three patients were only partly satisfied and two patients were not satisfied. One had pain while working/holding the steering wheel. The patients who were not satisfied were both treated with a screw fixation. All patients were able to return to their previous work occupation.

Bony union was achieved in all patients. The mean preoperative gap was 2.4 mm (SD 1.8, range 0.6–8.0 mm) and step was 1.0 mm (SD 1.1, range 0–2.9 mm). The mean postoperative gap was 0.1 mm (SD 0.8, range 0–2.7 mm) and step 0.6 mm (SD 0.7, range 0–2.0 mm). A joint subluxation was present in five patients preoperatively and none postoperatively. The extension angle on radiographs showed a mean value of 77° (SD 6.0°, range 67–88°).

Osteoarthritic changes on radiographs were seen in eight patients, however, five had arthritic changes already on the initial fracture radiographs. In most of the joints affected by osteoarthritis, a narrowed joint space as well as osteophytes could be detected. In contrast, no debris cysts were found in any patient.

The presence of postoperative OA nor IP joint transfixation did not show an impact on any outcome parameter (*p* = ns). However, patients with radiologic signs of OA showed a significantly higher postoperative gap [*p* = 0.021/0.4 mm (SD 0.5 mm) vs. 1.4 mm (SD 0.8 mm)].

There was no difference in the outcome parameters between patients treated with open or closed reduction (*p* = ns) except for a worse DASH score [*p* = 0.031/20 (SD 20) vs. 2 (SD 3)], a PRWE score [*p* = 0.023/19 (SD 20) vs. 2 (SD 4)] and MWS score [*p* = 0.008/72 (SD 12) vs. 86 (SD 4)].

There was no difference in any outcome parameter between blue collar workers, white collar works and retirees (*p* = ns) except a lower Kapandji grade in blue collar workers [*p* = 0.05/6.8 (SD 3.5) vs. 9.6 (SD 0.5) vs. 8.7 (SD 0.6)].

Hardware removal was performed in thirteen patients (81%) after a mean of 31 days (SD 6.5, range 18–43 days) including all patients with K-wires and one with a plate fixation.

Wound healing was satisfactory in all patients except one showing a small wound necrosis in the beginning after a closed fracture but ending up unspectacularly after conservative treatment (open reduction and screw fixation), yet with a worse clinical outcome than others. One patient with an open direct K-wire fixation and tendon suture developed a nail growth disturbance after an infection which was treated by antibiotics solely. Another patient who developed a documented infection with a local swelling and redness was also treated with antibiotics satisfactory (indirect and direct K-wire fixation). One patient developed a prolonged nausea (anchor fixation with K-wire transfixation) and another one prolonged pain due to a too tight cast application (screw fixation with a K-wire transfixation). There were no case of implant failure, neurovascular injury or crepitations. The overall complication rate was 31% (5/16 patients). One patient who was not included in the follow-up, suffered from an infection and osteomyelitis after an indirect K-wire fixation, which lead to a thumb amputation. All patient data and outcome results are presented as supplementary material in Tables 3, 4, 5 and 6.

### Literature search

**Fig. 2:**
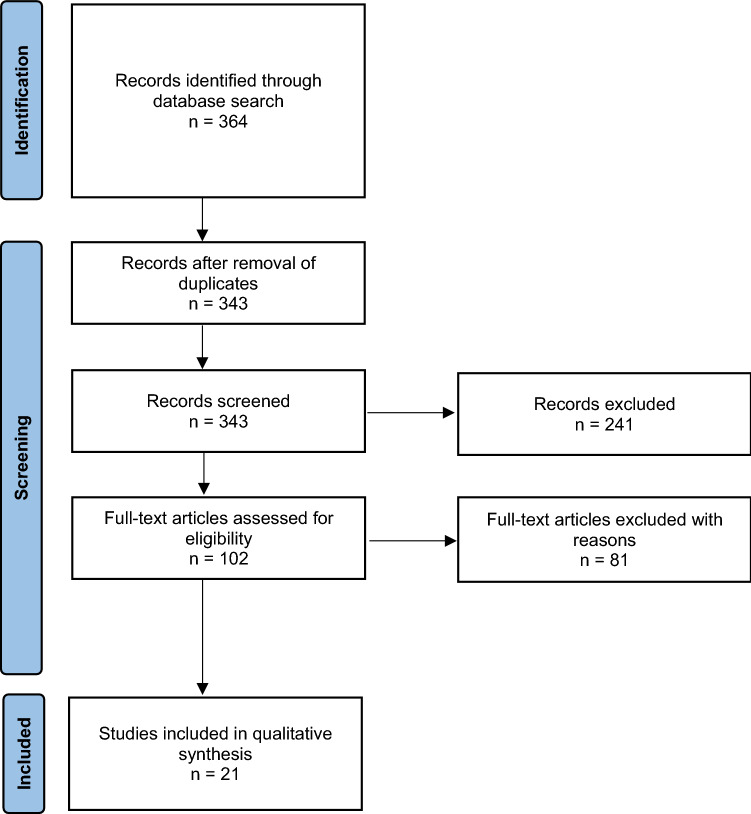
PRISMA flow diagram showing the study selection process

The literature selection process was conducted in accordance to the PRISMA guidelines [26] and is shown as a flow diagram in Fig. 2. Table 2 presents the detailed study characteristics, follow-up period, patient count of included bony mallet thumbs, injury mechanism, fracture morphology, treatment method, postoperative treatment method, patient outcome and complications of all included studies.

**Table 2:** Detailed study characteristics of all included studies sorted by the treatment method

Author	Year	Study type	Follow-up in months	Patient count*	Injury mechanism	Fracture morphology	Treatment method	Postoperative treatment	Outcome^†^	Complications^‡^
Nonoperative and inhomogenous treatment										
Kalainov et al. [15]	2005	Retrospective study	24.5	1/22	n.s.	33–66% articular surface and/or subluxation	Palmar thermoplastic splint for 5.5 ± 1.2 weeks	n/a	n.s./full bony union; no skin abnormality or nail-plate deformity	n.s./complication rate 9%; transient skin irritation (*n* = 2)
Oflazoglu et al. [31]	2017	Retrospective study	50 (6–161)	24	Cut with table saw (*n* = 1); Fall (*n* = 7); Sport tennis (*n* = 2), basketball (*n* = 3), flag football (*n* = 1), ice hockey (*n* = 1), football (*n* = 1); Playing with son (*n* = 1); catching a bottle (*n* = 1); jammed thumb (*n* = 2); suicide attempt (*n* = 1); unspecified (*n* = 3)	39% (10–69%) articular surface involvement; 26% (0–89%) fragment displacement; no subluxation; open fracture (*n* = 1)/closed fracture (*n* = 23); comminuted fracture(*n* = 2)	Splint fixation (*n* = 12); Cast fixation (*n* = 4); K-wire fixation (*n* = 3); Screw fixation (*n* = 2); Suture fixation (*n* = 1); Unknown (*n* = 2)	Inhomogenous: spica/short splint/splint/night splint/cast/opponens splint for a total duration of 4–12 weeks	n.s.	Complication rate 8.3%; wound complication (*n* = 1) K-wire fixation in a patient who cut himself with a table saw); prominent hardware (*n* = 1/screw fixation)
K-wire fixation										
Yamanaka and Sasaki [43]	1999	Case series	3	2/16	n.s.	> 1/3 articular surface	Compression fixation pin (1–2 × 1.2 mm)	Thermoplastic splint (average 12 days with active range of motion thereafter); Pin removal after bony union	no pain; 0/0/50° and 15/0/85°	n.s./rupture of the extensor tendon (*n* = 1)
Hofmeister et al. [13]	2003	Retrospective study	18	3/24	n.s.	> 25% articular surface or subluxation	Extension block pinning (indirect or with additional direct 1.4 mm K-wire pinning)	Splint	n.s./full bony union; no tender dorsal prominences, osteomyelitis, skin sloughs	n.s./complication rate 21%; superficial pin-site infection resolved with oral antibiotics (*n* = 3); slight fragment displacement (*n* = 2)
Phadnis et al. [33]	2010	Retrospective study	13 (10–21)	?/20	n.s.	> 30% articular surface with subluxation	Open reduction, direct K-wire fixation (2 × 0.6 mm) and K-wire transfixation (1 × 1.25 mm)	Hardware removal after 6 weeks; no routine external splinting	n.s./full bony union; no subluxation, no signs of osteoarthritis; all returned to work;	n.s./complication rate 9%; superficial infection resolved with oral antibiotics (*n* = 1)
Kang et al. [16]	2012	Case series	12 (9–16)	1/16	Bicycle fall	Thumb case: 48% articular surface involvement without subluxation	Open reduction, multiple direct or indirect K-wire transfixation (0.7 mm) and tendon suture	Hardware removal after 4–6 weeks followed by 2 weeks of volar splinting	Thumb case: full joint extension, full flexion, no pain/full bony union;	Transient nail deformity (*n* = 3—including one thumb)/5–10° extension or flexion lag (*n* = 5)
Nishimura et al. [29]	2012	Case report	7	1	Bicycle accident	1/3 articular surface	Ishiguro extension block technique in IP flexion (1 × 1.4 mm and 1 × 1.2 mm)	K-wire removal after 4 weeks; coil-splint for another 2 weeks; (6 weeks immobilization)	16/0/94°, no difficulties at work or playing bass guitar	None
Mifune et al. [25]	2016	Case report	4	1	Basketball injury	> 50% articularsurfaceinvolvement	Two extension block K-wires technique (2 × 1.2 mm + 1 × 1.5 mm)	4 weeks immobilization	− 5/0/90° (contralateral side 0/0/90); no difficulties working or playing basketball; full bony union	None
Han et al. [9]	2018	Case series	6.5 (5–11)	1/41	n.s.	> 30% articular surface or subluxation	Direct pinning (1 × 0.9 mm K-wire + transfixaion K-wire)	Aluminium finger splint for 4–5 weeks	n.s./no cases of major infection or pin loosening	n.s./mild erythema (*n* = 7)
Screw fixation										
Groebli et al. [8]	1987	Case series	26 (8–51)	1/21	n.s.	60% articular surface	Screw fixation	n.s.	n.s./no patient stopped working; no cutaneous necrosis	n.s./complication rate 14%; dystrophic disorders of the pulp with hypoesthesia (*n* = 3)
Hiwatari et al. [12]	2012	Case series	4 (2–12)	3/43	n.s.	> 30% articular surface or subluxation or loss of joint congruity	Screw fixation (1.2 mm)	n.s./thermoplastic custom splint for 4 weeks in some patients	n.s./no infections, nail deformities or implant complications	None
Afshar et al. [2]	2021	Case report	18	1	Fall during soccer	50% articular surface; comminuted fracture;	Failed closed reduction percutaneous pinning attempt—open reduction and screw fixation (1 × 2.0 mm)	Spica cast for 6 weeks	0–0–45°, no nail deformity, full bony union	None
Plate fixation										
Thirumalai et al. [36]	2017	Retrospective study	3 months/?	1?/35/(63)	Fall	40% articular surface	Open reduction and hook plate fixation (1.3 mm)	Splint at rest for 4 weeks with mobilization from the beginning	No extensor lag, nail bed deformity or skin erosion; full bony union	None/implant removal in 40% (*n* = 14); complication rate 17%; nail deformity (*n* = 6); plate extrusion (*n* = 5)
Vester et al. [40]	2018	Retrospective study	n.s./3 to > 12	1/38	n.s.	> 20% articular surface	Hook plate fixation	n.s.	Self-assessment score 26.3 (0 best/10 worst); SF-36 Score 50.22	n.s./implant removal in 37% (*n* = 14)
Xiong et al. [42]	2019	Case series	5 ± 4	2/157	n.s.	12–67% articular surface	Transverse two hole mini-plate (1.7 mm) with K-wire transfixation (0.9 mm)	K-wire removal after 2–6 weeks	n.s.; full bony union; no skin necrosis; no nail deformity	n.s./complication rate 9%; superficial infection (*n* = 1); skin irritation (*n* = 5); asymptomatic joint step (*n* = 8)
Mukasa et al. [27]	2019	Case report	6	1	Softball injury	30–50% articular surface	Hook plate technique (1.5 mm)	1 week splint and IP joint motion thereafter; hardware removal after 3 months	− 8/0/80 (contralateral side 10/0/85); full bony union; no difficulties at work or playing softball	Gradually improving transverse line nail deformity
Wire fixation										
Bischoff et al. [4]	1994	Case series	14	4/51	n.s.	> 25% articular surface and/or subluxation	Tension band fixation (28-gauge stainless steel wire)	n.s./dorsal aluminium splint for 5 weeks followed by another 5 weeks night splinting	n.s./delayed union (*n* = 1), non-union (*n* = 3); inability to return to work (*n* = 4); narrowing of the joint space (*n* = 20); persistent subluxation (*n* = 10), articular step (*n* = 8); overall 21 poor results, 20 satisfactory and 10 excellent results	n.s./complication rate 47%; superficial infections (*n* = 3), deep infections (*n* = 3), secondary fragment displacement (*n* = 3), fragment resorption (*n* = 4), secondary tendon rupture (*n* = 1), avascular necrosis (*n* = 1), nail growth disturbance (*n* = 6); implant removal mainly due to complications in 39% (*n* = 20)
King et al. [17]	2001	Retrospective study	16 (12–30)	1/24/(59)	n.s.	> 33% articular surface with or without subluxation	Open reduction, nylon suture (40) or pull-out wire (24-gauge) suture with K-wire transfixation (1 × 1.1 mm)	Pull-out wire suture removal after 4 weeks, transfixation K-wire removal after 6 weeks	n.s.	n.s./complication rate 41%; transient marginal skin necrosis (resolved after ~ 2 weeks/1 case required debridment after an open injury); recurrent mallet deformity within 8 weeks (*n* = 8); pin tract infection (*n* = 4); osteomyelitis (*n* = 2); nail deformity (*n* = 4); hardware breakage (*n* = 1); premature epiphyseal plate closure (*n* = 1); radial joint deviation (*n* = 1); joint hypertrophy (*n* = 1)
Zhang et al. [44]	2010	Case series	25.5 (24–27)	2/65	n.s.	n.s./30–49% articular surface	Pull-out wire (28-gauge) fixation with K-wire stabilization (0.9–1.1 mm)	Hardware removal after bony union (mean 46 days, 42–53 days)	n.s./surgically reduced fracture fragment maintained in all cases; no skin necrosis, break-down or infection; no nail deformity nor prominence	None
Biodegradable device fixation										
Nelis and Wouters [28]	2008	Case series	2	3/9	2 × work 1 × sports	> 1/3 articular surface or subluxation	Biodegradable device/Meniscu s Arrow^®^ (1.1 mm)	4 weeks mallet splint	n.s./all patients satisfied with full bony union; no nail bed deformities or dorsal bumps; all wounds healed; no secondary surgeries	None
Aarts et al. [1]	2014	Case series	4 (1–17)	5/50	n.s.	> 30% articular surface	Biodegradable device/Meniscus Arrow^®^ (1.1 mm)	Mallet splint for 5 weeks followed by 3 weeks with the splint during activities	n.s./full bony union; no skin abnormalities except local tender soft tissue swelling for 12 weeks (*n* = 2)	n.s./complication rate 6%; superficial infection resolved with oral antibiotics (*n* = 1); implant migration after 3 and 6 months with consecutive implant remnants removal (*n* = 2)

*n.s.* not specified, *n/a* not applicable, *IP* interphalangeal

*Bony mallet thumb fractures/all patients including bony mallet finger fractures

^†^Outcome of bony mallet thumb fractures/outcome of all included patients

^‡^Complications of bony mallet thumb fractures/complications of all included patients

All studies show a low level of evidence. They are either case reports, case series or they mix the outcome of the mallet thumb fracture patients with mallet finger fracture patients. There are many different surgical treatment methods, implants and implant sizes and postoperative immobilization regimes. The follow-up is mainly very short and the final outcome and the complications are scarcely reported when regarding the thumb in isolation. The overall infection rate was 0% for non-operative treatment, 3.4% for K-wire fixation methods, 0% for screw fixation methods, 0.4% for plate fixation methods, 8.6% for wire fixation methods and 2% for the fixation method using a biodegradable device (Table 2).

## Discussion

This study revealed that the evidence for the best treatment of a mallet thumb fracture is very low. Although some case reports showed a good clinical outcome using different kind of fixation methods, our case series, with the highest number of patient, including a detailed outcome report, revealed that the range of motion and strength is significantly inferior compared to the contralateral side. Some patients were unsatisfied and achieved a bad outcome with significant pain while working and a reduced range of motion.

Drilling across a finger joint can lead to heat osteonecrosis and destruction of the joint [3, 7, 37], however we did not observe this complication. An IP joint transfixation had also no impact on the clinic outcome. Therefore, it should be used if need for a stable fixation, for example for an indirect K-wire fixation; however, it is not necessary in cases of a stable screw or plate fixation. It can safely be used in cases of doubt as it does not negatively influence the outcome. The size of the transfixation K-wire can range between 0.9 and 1.5 mm (Table 2) without any evidence of any impact on the clinical outcome.

Although blue collar workers are more dependent on their hands while working compared to white collar workers and retirees, this study could not find any difference in the clinical outcome except for a worse Kapandji grade. The lower opposition grade did not seem to influence their functional scores, pain level or IP joint motion or limit their working capacity. However, the case load per group was low and this finding could be accidentally. A similar outcome for blue and white collar workers was also seen after traumatic brain injuries, while retirees had a significantly worse outcome [34]. In contrast, a fact that we could not investigate was that blue collar workers had a significantly longer duration of sick leave than white collar workers after a carpal tunnel release which may possibly be explained by the fact that these patients have a burdensome job for their hands and that they need more time for recovery [32].

An important finding was that open reduction seemed to have lead to a worse clinical outcome regarding the functional scores than a closed reduction technique, despite a similar range of motion, strength and pain level. Other studies showed a similar clinical outcome between an open reduction using a screw or plate fixation and a closed reduction using a K-wire fixation in proximal phalanx fractures, while others found a worse range of motion and grip strength in the closed reduction group [11, 19]. Because the functional scores were worse in the open approach group in our study, it seems advisable that the surgeon should try to perform a closed reduction whenever possible and feasible to achieve the best clinical outcome with regard to the patient’s risk for an infection. However, there was no difference in the overall outcome between patients being treated with K-wires compared to other treatment procedures in this study. Certainly a plate fixation method or screw fixation method needs an open approach, yet the approach may possibly be limited to a small mini-open entry for the implant leaving the two dorsal veins intact to possibly lower the postoperative swelling.

Although this study found a worse outcome of functional scores in patients treated with an open approach, a difference in the surgical method could not be seen. Nor literature nor our data could see an objective superiority of one method over another. All methods seem to have their legitimacy in the treatment of a bony mallet thumb fracture. But each surgical method can lead to specific complications. Namely, superficial infections are reported to occur in K-wire fixation, plate fixation, tension band fixation and biodegradable device fixation techniques [1, 4, 13, 33, 42]. Usually, this complication, however, resolved with oral antibiotics but the duration of administration is not reported sufficiently to minimize the risk of osteomyelitis. Osteomyelitis can occur and even lead to a thumb amputation as reported in one of our patients. In contrast to the long fingers, a thumb amputation has a significant impact on the overall hand function and should be avoided whenever possible. Therefore, a treatment method with the lowest infection rate should be selected, especially in high risk patients like patients suffering from diabetes, chronic alcohol consumption or low compliance. Thus, K-wire and wire fixation methods should rather not be used regarding this major complication.

Another common complication is the appearance of nail deformities, which especially occurs in tension band or pull-out wire fixation, plate fixation and rarely K-wire fixation methods. Some nail deformities may be due to the trauma itself, others may be due to an injury to the nail plate especially in an open approach by the surgeon. To minimize this complication closed techniques or the usage of screw fixation methods should rather be used than wire or plate fixation techniques.

Including all reported complications (including bony mallet finger fractures) of the published studies (Table 2) the overall complication rate ranges between 0 and 47% with minor and more severe complications. Our case series showed a rate of 31%, however, no patient needed a revision surgery. In cases of a complication implant removal is usually necessary [4, 36]. This study revealed an implant removal rate of 81%, however, K-wire removal is usually necessary by nature and was included leading to this high number. Screw fixation methods usually don’t need an implant removal, however, plate fixation methods can lead to a removal rate of up to 40% [36], or in our case series in one out of one cases (100%), mainly because of nail deformity problems.

There is also no standardized postoperative treatment regime. K-wire fixations need usually an immobilization for 4–6 weeks, however, some authors do not routinely use external splinting [33]. Although the authors did not see an implant failure, the K-wire can potentially break leading to a difficult surgery to remove both broken ends. More stable constructs (plate fixation, compression pin fixation) without IP joint transfixation can successfully be mobilized early after 1–2 weeks postoperatively [27, 36, 43]. However, a better or worse outcome with early mobilization cannot be deduced from the present data.

Nonunion seems to be very rare and only occurred in cases of a tension band fixation method [4]. Usually full bony union is achieved independent of all other fixation methods. Interestingly, this case series saw a high rate of postoperative radiologic signs of osteoarthritis. However, this did not correlate with the clinical outcome or pain which is similar to other joints [10, 24, 35].

The indication for surgery seems more or less homogenous across all reported studies. The involvement of 25–33% of the articular surface is the main indication as well as the presence of a joint subluxation. Because data is often mixed with bony mallet finger fractures, the presence of IP joint subluxation is difficult to deduce from literature. Some authors did not see any subluxation in the IP joint including the highest case count of bony mallet thumbs (*n* = 24) [31], which is contrary to our findings which showed an IP joint subluxation in five patients (31%). This finding may potentially be explained by a different definition or measurement technique of an IP joint subluxation.

In total, data regarding a bony mallet thumb fracture are scarcely reported and often mix outcome and complication data with bony mallet finger fractures. Therefore, neither the systematic literature review nor our case series could lead to comprehensive recommendations for an optimal surgical procedure to achieve an optimal patient outcome.

The need for surgical treatment for a good outcome is also not sufficiently clarified as some authors treated their patient successfully nonoperatively despite an articular involvement of more than one third of the articular surface [15, 31]. Another unanswered question is whether the size and presence of any postoperative gaps and steps influence the clinical outcome or predispose to symptomatic osteoarthritis. An intraarticular step of 2 mm is regarded as a risk factor for osteoarthritis and a worse outcome in distal radius fractures [18, 23]. It is unclear whether this limit is also true for the smaller finger joints and the IP joint of the thumb in particular. The mean postoperative gap was 0.1 mm and step 0.6 mm in our cohort, however, the range reached 2.7 mm and 2.0 mm, respectively. Yet, we did not see a significant difference in the clinical outcome in patients with higher graded gaps and steps. This is probably due to the low patient count especially in patients with higher graded gaps and steps.

The patient count of our case series is low and the treatment regime very inhomogenous which is a major limitation in our study limiting any statement on statistical significant differences. The low patient count is also true for the systematic review which is mainly limited to case reports regarding a bony mallet thumb fracture or data is pooled and mixed with bony mallet finger fractures. Therefore, a comprehensive treatment procedure leading to an optimal clinical outcome cannot be deduced. The main reason for the low patient count is the rare occurrence of a bony mallet thumb injury. Therefore, prospective high quality studies should be conducted in a multi-center set-up to establish the best treatment regime with the best patient outcome and lowest complication risk.

## Conclusion

In conclusion, this study revealed that the evidence for the best treatment of a bony mallet thumb fracture is low. There are many ways leading to Rome and to a successful and good clinical outcome for the patient. On one hand the functional outcome can be inferior using an open reduction approach, but on the other hand, a closed approach and K-wire fixation methods showed a higher risk for infection. Future multi-center research must be conducted to find the best treatment procedure for the best outcome of the patient.

## Supplementary Information

Below is the link to the electronic supplementary material.

Supplementary file1 (DOCX 15 kb)

Supplementary file2 (DOCX 14 kb)

Supplementary file3 (DOCX 13 kb)

Supplementary file4 (DOCX 12 kb)
