# Supplementary material for: Surgical treatment of the bony mallet thumb: a case series and literature review
Source: Arch Orthop Trauma Surg. 2022 Jan 15;142(5):887–900. doi: 10.1007/s00402-021-04333-w (PMC8994723; doi:10.1007/s00402-021-04333-w)
Supplement: Supplementary file 1 — Supplementary file1 (DOCX 15 kb) [file 402_2021_4333_MOESM1_ESM.docx]

| Table 6: Radiologic outcome parameters and complications | | | | | | | | |
| --- | --- | --- | --- | --- | --- | --- | --- | --- |
| Patient | Preop Gap in mm | PreopStep in mm | Postop Gap in mm | PostopStep in mm | Extension angle | OA preop | OA postop | Complications |
| 1 | 1,5 | 0 | 0 | 0 | 79,5 | N | N | N |
| 2 | 2 | 0 | 1,9 | 0 | 70,9 | Y | Y | Infection |
| 3 | 1,4 | 0,7 | 0 | 0,8 | 81 | N | N | Prolonged pain due to tight cast |
| 4 | 2,3 | 0 | 0 | 0 | 80,5 | N | N | N |
| 5 | 1,5 | 0,9 | 0,9 | 0 | 75,2 | N | N | N |
| 6 | 8 | 2,5 | 1,7 | 1,3 | 87 | N | Y | N |
| 7 | 1,5 | 2,5 | 1 | 1,2 | 66,5 | N | N | N |
| 8 | 2,2 | 0 | 0 | 0,4 | 80,5 | N | N | N |
| 9 | 1,3 | 0 | 0,6 | 0 | 75,6 | Y | Y | Woundnecrosis |
| 10 | 1,4 | 2,1 | 0,4 | 0,7 | 72,9 | N | Y | N |
| 11 | 0,6 | 1,1 | 0 | 1,2 | 81,3 | N | N | N |
| 12 | 2,8 | n/a | 1,1 | 0 | 74,3 | N | Y | Infection and nail growth disturbance |
| 13 | 1,5 | 2,9 | 2,7 | 1,2 | 79,4 | Y | Y | N |
| 14 | 3,6 | 0 | 1,2 | 0 | 77,9 | N | N | Prolongednausea |
| 15 | 4,5 | 1,5 | 1,9 | 2 | 87,9 | Y | Y | N |
| 16 | 2,7 | 1 | 0,5 | 0 | 68 | Y | Y | N |
| OA = osteoarthritis;preop = preoperatively; postop = postoperatively; N = no; Y = yes | | | | | | | | |
