# Supplementary material for: Surgical treatment of the bony mallet thumb: a case series and literature review
Source: Arch Orthop Trauma Surg. 2022 Jan 15;142(5):887–900. doi: 10.1007/s00402-021-04333-w (PMC8994723; doi:10.1007/s00402-021-04333-w)
Supplement: Supplementary file 2 — Supplementary file2 (DOCX 14 kb) [file 402_2021_4333_MOESM2_ESM.docx]

| Table 4: Injury specifications and treatment method | | | | | | | | | | |
| --- | --- | --- | --- | --- | --- | --- | --- | --- | --- | --- |
| Patient | Fragment size in mm | Articular involvment in % | Fracture fragments | Open/Closed injury | Doyle Classification | Days until surgery | Type of treatment | Open/closed reduction | Transfixation | Splint duration in days |
| 1 | 9,0x3,8 and 3,4x1,4 | 55 | 3 | Closed | IVc | 1 | Indirect Ishiguro K-wire fixation | Closed | Yes | 28 |
| 2 | 6x4 | 55 | 2 | Closed | IVc | 1 | Indirect Ishiguro + direct K-wire fixation | Closed | Yes | 27 |
| 3 | 6,9x4 | 25 | 2 | Closed | IVb | 2 | Screw fixation + K-wire transfixation | Open | Yes | 25 |
| 4 | 6,4x5,3 | 60 | 2 | Closed | IVc | 3 | Hook plate fixation | Open | No | n/a |
| 5 | 1,6x3,8 and 4,2x5,8 | 45 | 3 | Closed | IVb | 0 | Direct K-wire fixation | Closed | No | 28 |
| 6 | 4x5,5 | 90 | 2 | Closed | IVc | 2 | Indirect Ishiguro + direct K-wire fixation | Open | Yes | 29 |
| 7 | 2,6x4,2 | 20 | 2 | Closed | IVb | 0 | Direct K-wire fixation + K-wire transfixation | Closed | Yes | 41 |
| 8 | 2,9x4 | 40 | 2 | Closed | IVb | 1 | Indirect Ishiguro K-wire fixation | Closed | Yes | 36 |
| 9 | 5x10 | 60 | 2 | Closed | IVc | 13 | Screwfixation | Open | No | 27 |
| 10 | n/a | 20 | multiple | Closed | IVb | 1 | Direct K-wire fixation (2 K-wires) | Open | No | 33 |
| 11 | 10,8x3,8 and 2,1x17,7 | 50 | 3 | Closed | IVc | 1 | Twoscrewfixation | Open | No | 28 |
| 12 | 4,8x8,6 | 90 | 2 | Open | IVc | 0 | K-wire transfixation (2 K-wires) | Open | Yes | 43 |
| 13 | 5,6x2,5 | 20 | 2 | Closed | IVb | 10 | Direct K-wire fixation (3 K-wires) | Closed | No | 43 |
| 14 | 1x2,9 | 5 | 2 | Closed | I | 4 | Anchor fixation + K-wire transfixation | Open | Yes | 51 |
| 15 | 8,9x 7,0 and 8x7x2,6 | 40 | 3 | Closed | IVc | 4 | Indirect Ishiguro + direct K-wire fixation | Closed | Yes | 44 |
| 16 | 7x10 | 60 | 2 | Open | IVc | 18 | Screw fixation (2 screws) | Open | No | 27 |
