# Supplementary material for: Surgical treatment of the bony mallet thumb: a case series and literature review
Source: Arch Orthop Trauma Surg. 2022 Jan 15;142(5):887–900. doi: 10.1007/s00402-021-04333-w (PMC8994723; doi:10.1007/s00402-021-04333-w)
Supplement: Supplementary file 3 — Supplementary file3 (DOCX 13 kb) [file 402_2021_4333_MOESM3_ESM.docx]

| Table 5: Clinical outcome parameters | | | | | | | | | | | | | |
| --- | --- | --- | --- | --- | --- | --- | --- | --- | --- | --- | --- | --- | --- |
| Patient | Extension | Flexion | Total rangeofmotion | Kapandjigrading | Tippinch | Lateral keypinch | Gripstrength | Painunderload | DASH | PRWE | MWS | Buck Gramcko | Satisfaction |
| 1 | 36 | 67 | 103 | 10 | 3,5 | 6,4 | 27,8 | 0 | 0 | 0 | 90 | 13 | S |
| 2 | 32 | 26 | 58 | 9 | 1,6 | 3,3 | 18,3 | 0 | 3,3 | 2,5 | 85 | 11 | S |
| 3 | 9 | 26 | 35 | 9 | 6,7 | 3,7 | 35,1 | 4 | 27,5 | 25 | 75 | 6 | NS |
| 4 | 45 | 68 | 113 | 10 | 4,4 | 8,7 | 47,1 | 0 | 1,7 | 6,5 | 80 | 15 | S |
| 5 | 18 | 86 | 104 | 8 | 7,3 | 12,8 | 62,3 | 0 | 0 | 0 | 90 | 15 | S |
| 6 | 21 | 60 | 81 | 9 | 6,1 | 6,8 | 42,5 | 0 | 0,8 | 0 | 80 | 15 | S |
| 7 | 21 | 60 | 81 | 9 | 3,8 | 5,6 | 38,6 | 0 | 0 | 0 | 80 | 15 | S |
| 8 | 25 | 57 | 82 | 6 | 3,5 | 5,2 | 33,2 | 2 | 6,7 | 2 | 80 | 14 | S |
| 9 | 11 | 31 | 42 | 1 | 2,1 | 2,9 | 14,8 | 5 | 50 | 61 | 60 | 12 | NS |
| 10 | 0 | 32 | 32 | 10 | 5,4 | 9,2 | 45 | 4 | 15 | 5,5 | 70 | 8 | S |
| 11 | 27 | 94 | 121 | 10 | 6,8 | 7,5 | 52,4 | 0 | 0 | 1,5 | 90 | 15 | S |
| 12 | 0 | 32 | 32 | 10 | 2,2 | 4,2 | 26,3 | 1 | 11,7 | 14,5 | 70 | 9 | PS |
| 13 | 29 | 72 | 101 | 9 | 5 | 8,4 | 42,1 | 1 | 5 | 12 | 85 | 15 | PS |
| 14 | 24 | 33 | 57 | 8 | 0,6 | 2 | 3,7 | 4 | 53,3 | 40,5 | 50 | 12 | PS |
| 15 | 31 | 68 | 99 | 10 | 8,1 | 9 | 52,6 | 1 | 0 | 0 | 90 | 15 | S |
| 16 | 15 | 53 | 68 | 9 | n/a | n/a | 27,8 | 1 | 21,7 | 20 | 75 | 15 | S |
| S = satisfied; PS = partly satisfied; NS = not satisfied | | | | | | | | | | | | | |
