# Supplementary material for: Surgical treatment of the bony mallet thumb: a case series and literature review
Source: Arch Orthop Trauma Surg. 2022 Jan 15;142(5):887–900. doi: 10.1007/s00402-021-04333-w (PMC8994723; doi:10.1007/s00402-021-04333-w)
Supplement: Supplementary file 4 — Supplementary file4 (DOCX 12 kb) [file 402_2021_4333_MOESM4_ESM.docx]

| Table 3: Patient demographics | | | | | | | | | |
| --- | --- | --- | --- | --- | --- | --- | --- | --- | --- |
| Patient | Sex | Age at trauma | Profession | Follow-up in months | Mechanism | Affected Side | Dominant Side | ContralateralInjury | Subluxation |
| 1 | F | 15 | White collarworker | 132 | Moped crash | L | R | N | N |
| 2 | M | 78 | Retiree | 84 | Fall | L | R | Y (Bennett Fracture) | N |
| 3 | M | 43 | White collarworker | 41 | Not able to recall | L | R | N | N |
| 4 | M | 47 | White collarworker | 36 | Fall | L | A | N | N |
| 5 | M | 24 | Blue collarworker | 34 | Hit by an iron pipe | L | R | N | N |
| 6 | M | 18 | Blue collarworker | 27 | Bicyclecrash | L | R | N | N |
| 7 | M | 27 | White collarworker | 168 | Bicyclecrash | L | R | N | Y |
| 8 | M | 15 | Blue collarworker | 122 | Hit by soccer ball | L | R | N | Y |
| 9 | M | 59 | Blue collarworker | 17 | Thumb gotcaught | L | R | N | N |
| 10 | M | 16 | Blue collarworker | 124 | Hit by tombstone | L | R | N | N |
| 11 | M | 21 | White collarworker | 111 | Snowboard fall | R | R | N | N |
| 12 | F | 54 | White collarworker | 95 | Hit by board | L | R | N | Y |
| 13 | M | 42 | White collarworker | 112 | Motorcyclecrash | R | R | Y (Fracture of the 5th digit; Lateral malleolar fracture) | Y |
| 14 | F | 68 | Retiree | 95 | Hiking fall | L | R | N | N |
| 15 | M | 47 | White collarworker | 174 | Skiing fall | R | R | N | Y |
| 16 | M | 68 | Retiree | 70 | Hiking fall | L | R | N | Y |
| M = male; F = female; R = right; L = left; N = no; Y = yes | | | | | | | | | |
